# Association of the Sialylation of Antibodies Specific to the HCV E2 Envelope Glycoprotein with Hepatic Fibrosis Progression and Antiviral Therapy Efficacy

**DOI:** 10.1155/2020/8881279

**Published:** 2020-06-23

**Authors:** Oleg Kurtenkov, Jelena Jakovleva, Boris Sergejev, Julia Geller

**Affiliations:** Department of Virology and Immunology, National Institute for Health Development, Hiiu 42, Tallinn 11619, Estonia

## Abstract

The E2 envelope glycoprotein of the hepatitis C virus (HCV) is a major target of broadly neutralizing antibodies that are closely related to a spontaneous cure of HCV infection. There is still no data about the diversity of E2-specific antibodies (Abs) glycosylation. The aim of this study was to analyze the level and sialylation of E2 IgG Abs, the relation of the respective changes to hepatic fibrosis (F) progression and their possible association with the efficacy of interferon-*α*-2a plus ribavirin (IFN-RBV) antiviral therapy. One hundred three HCV infected treatment-naive patients were examined using ELISA with E2 recombinant protein as antigen and sialic acid-specific Sambucus nigra agglutinin. The efficacy of the IFN-RBV treatment of patients with HCV dominant 1b and 3a genotypes (GT) was evaluated. A significant decrease of E2 Abs sialylation in the late stages of fibrosis was found irrespective of HCV genotype. On this basis, the F4 stage of fibrosis can be discriminated from its F0 or F1-3 stage by an about 75-79% accuracy. HCV infection of 1b genotype is associated with the production of lower sialylated E2 Abs, a higher frequency of F4 stage fibrosis, and a worse response to antiviral therapy. The increased SNA reactivity of E2 Abs was observed in patients with a sustained virological response (SVR). The proportion of SVR responders was significantly higher among patients with 3a genotype. However, for both dominant HCV genotypes (3a and 1b), an increased sialylation of E2 IgG was associated with a higher rate of patients with sustained virological response to antiviral therapy. Thus, the association of alterations of anti-E2 IgG Abs sialylation with hepatic fibrosis stage, HCV genotype, and the efficacy of antiviral therapy enables using these changes as novel noninvasive predictive biomarkers. The clinical potential of these findings is discussed.

## 1. Introduction

Hepatitis C virus (HCV) is a serious health problem worldwide and patients with this infection remain a difficult-to-cure population [1, 2]. HCV envelope glycoproteins E1 and E2 play an important role in the HCV entry process, and the tetraspanin CD81 is the best-characterized entry factor which has been shown to interact with the HCV E2 envelope glycoprotein [3–5]. The E2 (gp70) is one of the main targets of the humoral immune response against HCV and also a major target of broadly neutralizing antibodies against HCV [6–8]. Apart from its high genetic heterogeneity, HCV has developed various ways to escape the host immune response. One of them is the protection by a glycan shield that masks conserved neutralizing epitopes at the HCV surface, thus evading immune detection [9, 10]. Many studies have been focused on the antigenic face of E2, its modification by glycan shifting, and the potential role of E2 in vaccine design [5, 7, 8, 11–14]. The important role of antienvelope Abs response in the resistance to and a spontaneous cure of HCV infection has been demonstrated [15–17]. However, the structural heterogeneity of E2-specific Abs (E2 Abs), in particular, their glycosylation profile, and its possible impact on the efficacy of antiviral therapy still remain largely unexplored.

There is strong evidence that Abs glycosylation may dramatically modulate their functioning and Fc-mediated effector mechanisms [18–23]. The glycodiversity of Abs is now a topic of interest because of the possibility to construct their glycoforms with the predicted potential, thus improving the immunotherapy efficacy [24, 25]. The diverse clinical effect of different Ab glyco-subsets has been demonstrated in autoimmunity, infections, and cancer [26–31]. The increased reactivity of total serum IgG and anti-*α*Gal IgG with several fucose-specific lectins was established in HCV-infected patients with cirrhosis [32]. However, it should be taken into account that the glycosylation of total serum IgG may significantly differ from that of antigen-specific IgG [33–35]. Thus, the determination of the total serum Abs glycosylation does not reflect the glycosylation profile of antigen-specific Abs. This implies that the glycosylation pattern of Abs against the target antigens involved in the pathogenesis of a specific disease may be more informative.

The main aim of this investigation was to analyze the level and sialylation of E2 IgG Abs, the relation of the respective changes to hepatic fibrosis progression, and their possible impact on the efficacy of (IFN-RBV) antiviral therapy.

In the present study, a significant decrease of E2 Abs sialylation was observed in the late stages of fibrosis (F), irrespective of HCV genotype (GT), and viral load level. These changes showed a rather high level of sensitivity and specificity (above 75%). Compared to 1b GT, a significantly higher E2 Abs sialylation was detected in patients infected with HCV 3a genotype. However, for both dominant HCV genotypes (3a and 1b), an increased sialylation of E2 IgG was associated with a higher rate of patients with a sustained virological response (SVR) to antiviral therapy. The possible clinical value of these findings is discussed.

## 2. Material and Methods

### 2.1. Subjects

Serum samples were taken from one hundred three HCV infected treatment-naive patients: men-66, women-37, median age-36 (range 19-58) years. Characteristics of patients by HCV genotype and the effect of antiviral therapy are presented in Table 1. The investigation was carried out in accordance with the ICH GCP Standards and approved by the Tallinn Medical Research Ethics Committee, Estonia. A written informed consent was obtained from all patients.

The diagnosis of HCV infection was based on the presence of anti-HCV antibodies in the sera, detection of serum HCV RNA, histologically verified fibrosis stage, and clinical follow-up. The HCV genotype was determined by the hybridization technique using VERSANT HCV genotype assay (LiPA) (Bayer HealthCare LLC, Tarrytown, NY). The range of fibrosis was classified according to the Metavir scoring system from F0 to F4 (cirrhosis). The serum samples were stored in aliquots at -40°C until use.

HCV infection treatment (pegylated interferon-*α*-2a plus ribavirin therapy) (IFN-RBV) was conducted according to the National Guidelines approved by the Estonian Society for Infectious Diseases in 2010. The Peg-IFN *α*-2a (Pegasys, F-Hoffmann La Roche Ltd, Basel, Switzerland) was administrated at a dosage of 180 g/week. The RBV (Copegus, (F-Hoffmann La Roche Ltd, Basel, Switzerland) was given per os at a dosage of 1,200 mg/day or 1,000 mg/day depending on body weight for patients with genotype 1b, and at a dosage of 800 mg/day in patients with genotype 3a. The response to therapy was evaluated as a sustained virological response (SVR) defined as serum HCV RNA undetectable at week 24 after the end of therapy; nonresponders (NR), i.e., patients in whom sera HCV RNA levels remained stable during treatment and relapses (RL)—patients who sero-reverted to HCV RNA during follow-up.

### 2.2. The E2 Glycoprotein Specific IgG Antibody Assay

The level of anti-E2 IgG was determined by the enzyme-linked immunosorbent assay (ELISA). The plates (NUNC Maxisorp, Denmark) were coated with an E2 recombinant protein (ViroStat, ME, USA) 5 *μ*g/ml in the carbonate buffer, pH 9.6. After the overnight incubation, triple washing and blocking with a Superblock solution (Pierce, USA) for 30 min at 25°C, the serum samples diluted 1 : 25 in PBS-0.05% Tween (Tw) were applied for 1.5 h at 25°C. After the subsequent washing with PBS-Tw, the level of bound anti-E2 Abs was determined using the alkaline phosphatase (AP) conjugated goat antihuman IgG, (Sigma, USA) and p-nitrophenylphosphate disodium hexahydrate (pNPP, Sigma, USA). The absorbance values were read at 405 nm (Tecan Reader, Austria). The optical density value (O.D.) of control wells (blank: the Superblock solution instead of serum) was subtracted from that of Ab-coated wells, and each sample was analyzed in duplicate. The interassay variations were minimized by including the standard serum and using the correction factor (CF): CF = 1/(standard serum A values–blank) × 100. The results were expressed in relative units (RU): RU = sample O.D.value × CF.

### 2.3. SNA Lectin Reactivity of E2 Antibodies

The lectin reactivity of E2-specific IgG antibodies was measured in a similar way, except that the binding of the neuraminic acid (sialic acid)-specific Sambucus nigra agglutinin (SNA) to the absorbed anti-E2 antibodies was determined. The biotinylated SNA (Vector Laboratories, Inc., USA) in 10 mmol/L Hepes, 0.15 mol/L NaCl, 0.1 mmol/L CaCl_2_, and pH 7.5 was applied at a concentration of 5 *μ*g/mL for 1.5 h at 25°C. The bound lectin was detected with a streptavidin-AP conjugate (Dako, USA) and pNPP (Sigma, USA). The O.D. of control wells (no serum sample) was subtracted from that of Ab-coated wells to determine the lectin binding. Each sample was analyzed in duplicate. The value of the SNA binding to E2-specific Abs and the ratio of SNA binding to E2-specific IgG value (SNA/IgG ratio) were determined.

### 2.4. Statistical Analysis

The results were analyzed using the Student *t*-test or nonparametric Mann-Whitney *U* test, where appropriate, the chi-square test and the ROC analysis. The respective difference between the groups was considered to be significant when *P* ≤ 0.05. All calculations were performed using the RStudio-1.1.463 software.

## 3. Results

**Figure 1:**
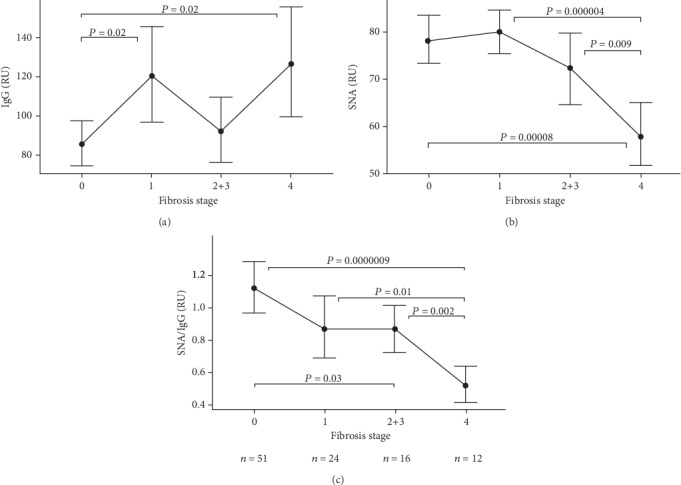
The level of anti-E2 IgG antibodies and their sialylation (SNA reactivity) by hepatic fibrosis stage (0-4).∗ (a) Anti-E2 IgG level. (b) SNA binding. (c) SNA binding/anti-E2 IgG level ratio. All parameters are presented in relative units (RU). The means and 95% confidence intervals are shown. *P* values are indicated for significant differences. ∗Stages 2 and 3 of fibrosis are combined due to the small number of patients (n =12 and 4, resp.).

**Figure 2:**
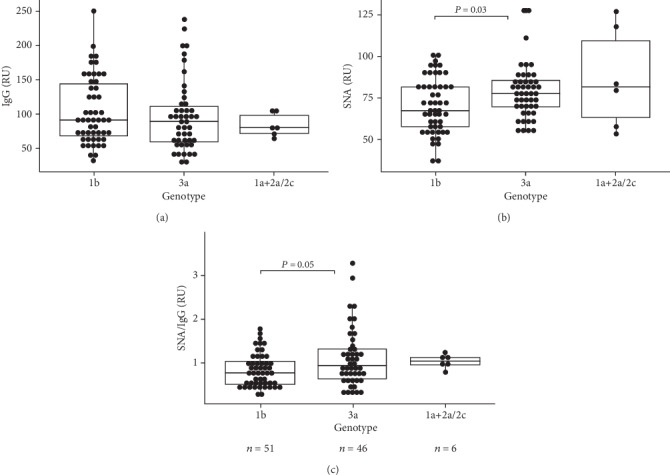
E2-specific IgG level and SNA reactivity by HCV genotype. (a) E2 IgG level. (b) SNA binding. (c) SNA/IgG ratio. Each dot represents one individual. Medians, ranges, and quartiles are shown, and *P* values are indicated for significant differences.

**Figure 3:**
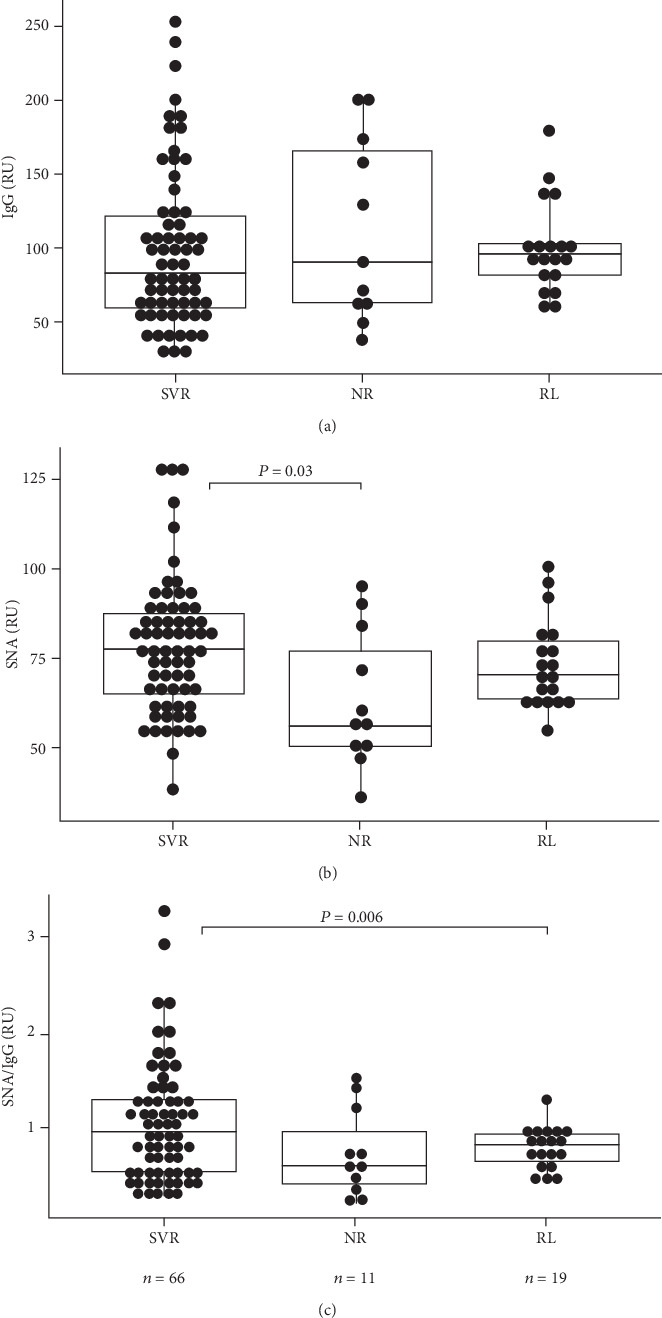
E2-specific Abs profile and IFN-RBV therapy efficacy. SVR: sustained virologic response; NR: no response; RL: relapse. Medians, ranges, and quartiles are shown, and *P* values are indicated for significant differences.

All the data obtained were analyzed by fibrosis stage, HCV genotype, viral load, and antiviral therapy effect. Compared to patients with no fibrosis (F0 stage), a moderate increase of E2 specific IgG was observed only in those with stage F1 (*P* = 0.017) and F4 (*P* = 0.01) (Figure 1(a)). In addition, no evident association between the level of E2 antibodies and HCV genotype or the efficacy of virotherapy was found (Figures 2(a) and 3(a)).

A dramatic decrease of E2 Ab SNA reactivity was found in the F4 stage of fibrosis (*P* = 0.00008), and this was also true for the SNA/IgG ratio (*P* = 0.00019) (Figures 1(b) and 1(c)). A significant decrease of this ratio (*P* = 0.03) was observed already in the early stages of fibrosis (F1-3), but the most uniform and pronounced decline of the ratio was noticed in the F4 stage of fibrosis (*P* = 0.0000009).

**Figure 4:**
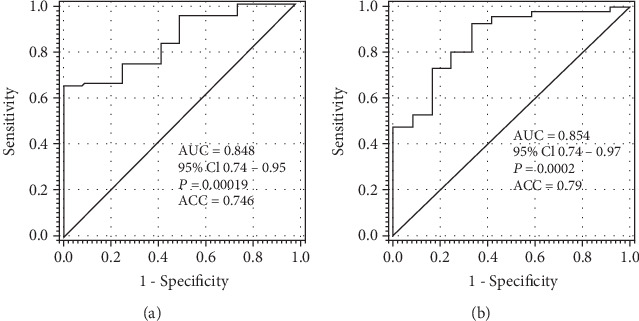
Sensitivity and specificity of anti-E2 IgG sialylation (SNA reactivity) changes in discriminating fibrosis stages F0 and F4 as evaluated by receiver operator characteristic (ROC) curve analysis. (a) SNA binding, stage F0 versus F4. (b) SNA/IgG ratio, stage F1-3 versus F4.

A rather high level of sensitivity and specificity of these changes for F4 versus F0 stage fibrosis (sensitivity-74.5%, specificity-75%) as evaluated by ROC analysis was achieved in the study (Figure 4(a)). Even better discrimination level was found between F4 and earlier stages of fibrosis (F1-3) with 80% sensitivity and 75% specificity, respectively, ACC value equal to 0.79 for SNA binding to E2 IgG (Figure 4(b)).

HCV 1b and 3a genotypes were dominant among the patients examined (Table 1). Only one patient with 1a genotype and five patients with 2a/2c genotype were detected in the study. The viral load values were very similar in patients with 1b and 3a genotypes (*P* = 0.41, data not shown).

Compared with F0 stage (no fibrosis), the viral load in fibrosis stage F1 was slightly increased but showed no difference from that of stage F4 (*P* = 0.97). No significant difference between 1a and 3a genotypes (*P* = 0.41, data not shown) was found either. Notably, among the patients investigated, the frequency of stage F4 fibrosis was about twice higher in patients with 1b genotype compared to those with 3a GT (15.7% and 8.7%, respectively) (Table 1).

Differences in the level of E2-specific IgG between 1b and 3a HCV genotypes were found to be insignificant (Figure 2). Compared with GT1b infected patients, a significantly higher SNA reactivity was demonstrated in patients with HCV 3a genotype. The respective values in patients with other genotypes were similar to those for 3a genotype patients. However, both genotype groups showed a significant decrease of Abs sialylation in stage 4 fibrosis compared with patients without fibrosis (F0) (Table 2).It has to be noted that the response of patients with 1b and 3a genotypes to virotherapy was quite different: the SVR rate was significantly higher in 3a genotype compared to 1b genotype: 80.4% and 47.1%, respectively (Table 1), (*χ*2 = 5.54, *P* = 0.018). Moreover, just the GT 3a SVR responders demonstrated a significantly higher SNA/IgG ratio compared to the 1b GT group (Table 3). A similar trend was noted for SNA binding values (*P* = 0.09). Most patients with NR or relapse showed a low level of E2 IgG SNA binding (*P* = 0.02) or SNA/IgG ratio (*P* = 0.006) (Figure 3).

The level of E2 IgG was not associated with the efficacy of antiviral therapy as evaluated by the proportion of patients with sustained virological response (SVR) (Figure 3(a)). However, the SVR group exhibited a significantly higher level of SNA binding to E2 IgG and SNA/IgG ratio compared to patients with “no response.” A similar trend was observed for the SNA/IgG ratio in patients with no response to therapy (*P* = 0.06). Patients with relapse exibited much lower SNA/IgG ratio values compared with the SVR group (*P* = 0.006).

Thus, the degree of E2-specific IgG sialylation may predict the hepatic fibrosis progression and sustained viral response to IFN-RV therapy.

## 4. Discussion

Antibodies to E2 HCV envelope glycoprotein are present in all HCV infected individuals and are closely related to broadly neutralizing Abs. The early production of bnAbs to E2 may contribute to controlling the virus, spontaneously curing the infection, and minimizing the masking of major conserved neutralizing epitopes with cross-reactive and interfering non-neutralizing Abs that usually appear later [6, 11]. Strikingly, in the noninvasive diagnosis of liver damage, very little attention is being paid to the diversity of HCV specific Abs, in particular to their glycosylation, though it is widely accepted that immune system-mediated reactions are involved in the HCV infection pathogenesis and outcome, including spontaneous HCV clearance [2, 17, 36, 37]. Since HCV is not cytopathic per se, the viral persistence and clinical outcome of the infection are likely due to variations in HCV capacity to escape the host innate and adaptive immunity.

The distribution of HCV genotypes in the present study was very similar to that reported previously for Estonia and some other European countries where GT 1 and GT3 account for the vast majority of HCV infections [38, 39]. A similar association of severe hepatic damage with the lower level of E2 Abs sialylation was observed in patients with both genotypes despite the higher sialylation of E2 Abs in patients with 3a GT and the fact that most patients with the late stages of fibrosis belonged to 1b GT group (10 of 14). Thus, changes in E2 Abs sialylation are a common phenomenon for both dominant genotypes. It has been reported that patients with 3a genotype infection have demonstrated a more aggressive HCV disease [2, 40, 41]. At the same time, it is just these patients whose proportion of SVR responders was significantly higher (80.4% versus 47.1% in 1b group, *χ*2 = 10.16, df = 1, *P* = 0.0014 (with Yates' continuity correction). Thus, it seems that the progression of the disease and the ability of the host to respond to antiviral therapy are the two quite different and obviously independent things. Our finding that the association of the increased E2 IgG sialylation is associated with a better efficacy of therapy supports the idea that more sialylated Abs demonstrate a higher anti-inflammatory potential. However, it remains unknown whether the higher sialylation of E2 Abs may promote HCV neutralizing activity, thus increasing antiviral therapy efficacy.

In developing countries, the Peg-IFN-a+RBV therapy still remains the principal treatment for patients with HCV [2]. However, a considerable percentage of patients with HCV genotype 1 infection does not respond to IFN-RBV therapy due to drug resistance and/or poor tolerability. In treatment with direct-acting antivirals (DAA), the SVR rates may exceed 80% irrespective of genotype though an interferon-based therapy may be still beneficial in DAA-resistant patients [40–43].

HCV has been involved in the triggering of autoimmune diseases with the development of mostly non-organ-specific autoantibodies (NOSA), and this was associated with a more severe histological and biochemical profile of hepatitis C infection [44–47]. Notably, the interferon therapy may also induce autoimmune disorders [46], while there is a negative correlation between the efficacy of antiviral treatment for HCV and the presence of NOSA [48]. Alterations of Abs glycosylation in autoimmunity a well-documented fact and the low level of IgG sialylation are usually associated with increased inflammation [22, 23]. The decreased level of sialylation of haptoglobin and alpha 1-antitrypsin was observed in HCV infected patients as evaluated by ELISA using SNA, whereas the increased level of sialylation was observed in hepatocellular carcinoma [49]. We have shown recently that the sialylation of Abs to tumor-associated Thomsen-Friedenreich glycotope is increased in gastric, breast, and colon cancer patients [28, 50, 51] and is associated with a worse survival rate of gastric cancer patients [28]. Using the lectin-based technology, Kuno et al. [52, 53] have noticed specific alterations of *α*1acid glycoprotein glycosylation that enable the monitoring of HCV infection progression. Total serum IgG galactose deficiency associated with liver damage was detected also in chronic hepatitis B (53). Changes in *α*Gal natural Abs (IgG) glycosylation (increased fucosylation) were observed in HCV-infected patients in the late stages of fibrosis [32].

Compared to HCV 1b genotype, a higher sialylation of E2 Abs in patients infected with HCV 3a genotype was found in the present study. Moreover, a better SVR rate was revealed in genotype 3a patients who demonstrated a higher SNA reactivity of E2 IgG antibodies. These findings allow us to suggest that these associations may be related to the well-known anti-inflammatory activity of higher sialylated Abs. It remains unknown whether HCV-infected patients with the low level of E2 Abs sialylation are a potential risk group for cirrhosis development, because we have no retrospective data about whether this group of patients revealed similar changes in the earlier stages of disease, before the F4 stage fibrosis development. However, it has to be considered that some patients with the low SNA binding or SNA/IgG ratio were present in every stage of the fibrosis, as well as in the F0 group (Table 1), suggesting that some infected individuals had initially a low level of Abs sialylation before the development of the late stages of the disease. Only a long-term follow-up of patients with the earlier stages of fibrosis (or without them) will give an answer to this question.

In conclusion, we provide evidence of the changes in the glycosylation of E2 specific Abs in HCV-infected patients. Novel findings of the association of anti-E2 IgG Ab 2,6 sialylation with the stage of HCV-induced hepatic fibrosis, HCV genotype, and the efficacy of antiviral therapy (IFN-RBV) are presented. HCV infection with 1b genotype is associated with the production of lower sialylated E2 Abs, a higher frequency of patients with F4 stage fibrosis, and a worse response to IFN-RBV antiviral therapy. Notably, not a level of E2 antibodies per se but rather their sialylation can be clinically useful for predicting liver damage and HCV infection treatment outcome. Whether this phenomenon is related only to E2-specific IgG and whether changes in E2 Abs sialylation may also be useful in DAA antiviral therapy prognosis remains to be determined. We realize that our findings need further clinical confirmation on a larger scale and also in patients treated with DAA. Future efforts should focus on the definition of HCV-specific Abs glycosubsets that could specifically modulate immune response to HCV, including HCV specific Abs neutralizing activity.

## Figures and Tables

**Table 1:** The characteristics of patients under investigation ∗.

HCV genotype (*n*)	Hepatic fibrosis stage				Response to treatment				
	0	1	2	3	4	SVR	NR	RL	ST
	*n* (%)	*n* (%)	*n* (%)	*n* (%)	*n* (%)	*n* (%)	*n* (%)	*n* (%)	*n* (%)
1b (51)	26 (51)	9 (17,6)	5 (9,8)	3 (5,9)	8 (15,7)	24 (47,1)	10 (19,6)	14 (27,5)	3 (5,9)
3a (46)	21 (45,7)	14 (30,4)	6 (13)	1 (2,2)	4 (8,7)	37 (80,4)	1 (2,2)	5 (10,9)	3 (6,5)
1a+2a/2c (6)	4 (66,7)	1 (16,7)	1 (16,7)	0 (0)	0 (0)	5 (83,3)	0 (0)	0 (0)	1 (16,7)
All cases (103)	51 (49,5)	24 (23,3)	12 (11,7)	4 (3,9)	12 (11,6)	66 (64,1)	11 (10,7)	19 (18,5)	7 (6,8)

∗Genotype 1a: one patient; GT 2a/2c: 5 patients. SVR: sustained virologic response; NR: no response, RL – relapse. In seven patients treatment was stopped (ST) due to intolerance.

**Table 2:** HCV E2 glycoprotein-specific antibody sialylation pattern in patients with F0 and F4 fibrosis stage by HCV genotype.

HCV genotype	SNA binding			SNA/IgG ratio		
	Mean ± SD		*P* value∗	Mean ± SD		*P* value∗
	F0	F4		F0	F4	
1b	72.40 ± 15.78	55.47 ± 14.27	0.01	0.96 ± 0.39	0.53 ± 0.23	0.0007
3a	81.69 ± 18.35	60.91 ± 6.88	0.002	1.31 ± 0.79	0.53 ± 0.24	0.002
*P* value	0.07	0.39		0.08	0.96	

∗*P* value between F0 and F4.

**Table 3:** Sialylation pattern of E2 IgG antibodies in patients with a sustained virological response by HCV genotype.

HCV genotype (*n*)	SNA binding	SNA/IgG ratio
	Mean ± SD	Mean ± SD
1b (24)	72.35 ± 16.87	0.84 ± 0.44
3a (37)	80.05 ± 16.94	1.18 ± 0.72
*P* value	0.09	0.02

## Data Availability

The data that support the findings of this study are available on request from the corresponding author.
